# Eriocalyxin B, a natural diterpenoid, inhibited VEGF-induced angiogenesis and diminished angiogenesis-dependent breast tumor growth by suppressing VEGFR-2 signaling

**DOI:** 10.18632/oncotarget.12652

**Published:** 2016-10-14

**Authors:** Xunian Zhou, Grace Gar-Lee Yue, Minghua Liu, Zhili Zuo, Julia Kin-Ming Lee, Mingyue Li, Stephen Kwok-Wing Tsui, Kwok-Pui Fung, Handong Sun, Jianxin Pu, Clara Bik-San Lau

**Affiliations:** ^1^ School of Biomedical Sciences, The Chinese University of Hong Kong, Shatin, New Territories, Hong Kong SAR, China; ^2^ Institute of Chinese Medicine, The Chinese University of Hong Kong, Shatin, New Territories, Hong Kong SAR, China; ^3^ State Key Laboratory of Phytochemistry and Plant Resources in West China, The Chinese University of Hong Kong, Shatin, New Territories, Hong Kong SAR, China; ^4^ State Key Laboratory of Phytochemistry and Plant Resources in West China, Kunming Institute of Botany, Chinese Academy of Sciences, Kunming, Yunnan, China

**Keywords:** Eriocalyxin B, angiogenesis, vascular endothelial growth factor (VEGF), vascular endothelial growth factor receptor 2 (VEGFR-2), breast cancer

## Abstract

Eriocalyxin B (EriB), a natural *ent*-kaurane diterpenoid isolated from the plant *Isodon eriocalyx* var. *laxiflora*, has emerged as a promising anticancer agent. The effects of EriB on angiogenesis were explored in the present study. Here we demonstrated that the subintestinal vein formation was significantly inhibited by EriB treatment (10, 15 μM) in zebrafish embryos, which was resulted from the alteration of various angiogenic genes as shown in transcriptome profiling. In human umbilical vein endothelial cells, EriB treatment (50, 100 nM) could significantly block vascular endothelial growth factors (VEGF)-induced cell proliferation, tube formation, cell migration and cell invasion. Furthermore, EriB also caused G1 phase cell cycle arrest which was correlated with the down-regulation of the cyclin D1 and CDK4 leading to the inhibition of phosphorylated retinoblastoma protein expression. Investigation of the signal transduction revealed that EriB inhibited VEGF-induced phosphorylation of VEGF receptor-2 via the interaction with the ATP-binding sites according to the molecular docking simulations. The suppression of VEGFR-2 downstream signal transduction cascades was also observed. EriB was showed to inhibit new blood vessel formation in Matrigel plug model and mouse 4T1 breast tumor model. EriB (5 mg/kg/day) treatment was able to decrease tumor vascularization and suppress tumor growth and angiogenesis. Taken together, our findings suggested that EriB is a novel inhibitor of angiogenesis through modulating VEGFR-2 signaling pathway, which could be developed as a promising anti-angiogenic agent for treatment of angiogenesis-related human diseases, such as cancer.

## INTRODUCTION

Angiogenesis is the formation of new capillaries from existing vessels, which is essential in a variety of physiological and pathophysiological processes including wound healing, granulation tissue formation, vascular disease and cancer [1–3]. Several sequential steps are involved, initiation is the first step by the dissolution of vascular basal membrane, which leads to the enhancement of vascular permeability and degradation of extracellular matrix, followed by endothelial cell migration, proliferation, invasion, which contributes to the new tube formation [4]. The newly formed vascular networks penetrate into tumors is responsible for nourishment supply and metabolic wastes removal in the tumor site, which will enable malignant tumor cells expansion, invasion, dissemination and subsequently metastasis [5]. Thus, anti-angiogenesis is now considered to be an effective strategy in anti-cancer therapy.

Vascular endothelial growth factors (VEGF) and the related receptors appear to be the key pro-angiogenic mediators in vascular development, including tumor neovascularization [6, 7]. Until now, three VEGF receptor tyrosine kinases (RTKs) have been identified, known as VEGFR-1 (Flt-1), VEGFR-2 (KDR/Flk-1) and VEGFR-3 (Flt-4). The role of VEGFR-1 on angiogenesis in endothelial cell remains unclear, although it has the highest affinity for VEGF but with much weaker kinase activity, which is not capable to generate any mitogenic signals [8–10]. VEGFR-2 is the primary regulator in VEGF-stimulated signal transduction, which is responsible for the control of endothelial cell survival, proliferation, migration, and invasion, etc [11, 12]. Thus, the suppression of VEGFR-2 signaling pathway has become a potential target in anti-angiogenesis therapies.

Over the last decade, several anti-angiogenic agents targeting on VEGF have been approved by FDA for the treatment of cancers, such as VEGF inhibitor bevacizumab (Avastin^®^), small-molecule RTK (receptor tyrosine kinase) inhibitor sorafenib (Nexavar^®^) and sunitinib (Sutent^®^), monoclonal antibody against VEGF receptor pazopanib (Votrient^®^) [13]. However, most of the available drugs have severe side effects (e.g. hypertension, proteinuria, thrombosis, bleeding, etc.). Therefore, there is still an urgent need for the development of new VEGF inhibitors targeting on angiogenesis with less side effects.

**Figure 1:**
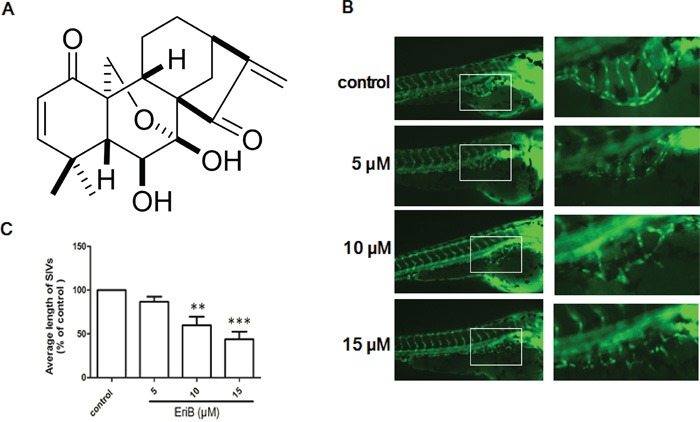
The inhibitory effects of EriB on the formation of subintestinal vessel (SIV) of Tg(fli1:EGFP) zebrafish embryos **A.** Chemical structure of EriB. **B.** The SIVs of zebrafish embryos treated with vehicle control (0.1% DMSO) developed into a smooth basket-like structure. Embryos treated with EriB (5, 10, 15 μM) for 72 h leads to an inhibition of SIV formation (magnification, ×100). **C.** The average length of SIVs was measured as described in materials and methods in total 80 zebrafish embryos. Each values was presented as means + SEM (n=80), **p < 0.01, ***p < 0.001 compared with control group (one-way ANOVA).

Recently, a variety of angiogenesis inhibitors from natural products targeting on VEGF and VEGFR-2 have been reported, such as 4-vinylphenol [14], deoxypodophyllotoxin [15], curcumin [16], gamabufotalin [17], ellagic acid [18], tryptanthrin [19], etc. Eriocalyxin B (EriB), a natural *ent*-kaurane diterpenoid isolated from *Isodon eriocalyx* var. *laxiflora* (family Lamiaceae) as shown in Figure 1A. Previous studies had revealed that EriB exhibited strong anti-leukemic activity and regulated inflammatory processes in lymphoma cells [20–22]. Besides, EriB also exerted anti-tumor activity in pancreatic cancer cells through the suppression of the glutathione and thioredoxin antioxidant systems [23, 24]. Additionally, the induction of apoptosis by EriB was observed in ovarian cancer cells and hepatocellular carcinoma cells via the inhibition of NF-kappa B signaling pathway [25, 26]. However, the potential influence of EriB on angiogenesis has not been elucidated.

In the present study, we found that EriB treatment was able to inhibit angiogenesis in zebrafish model, Matrigel plug assay and mouse 4T1 breast tumor model. The underlying mechanism involves the binding of EriB to the ATP-binding site of VEGFR-2, which results in the suppression of VEGFR-2 signaling pathway in human umbilical vein endothelial cells (HUVECs). Taken together, our findings suggested that EriB may be utilized as an anti-angiogenic agent for the treatment of angiogenesis-related diseases.

## RESULTS

### EriB diminished the subintestinal vessel (SIV) formation in zebrafish embryos

The vehicle-treated zebrafish embryos had normal vessel development, in which the SIVs formed as a smooth basket-like structure. When zebrafish embryos treated with EriB (5, 10 and 15 μM) for 72 h, the formations of SIVs were inhibited (Figure 1B). The average length of SIVs in zebrafish embryos were significantly diminished in a dose-dependent manner (Figure 1C).

### EriB altered the angiogenic genes expressions in zebrafish embryos

**Figure 2:**
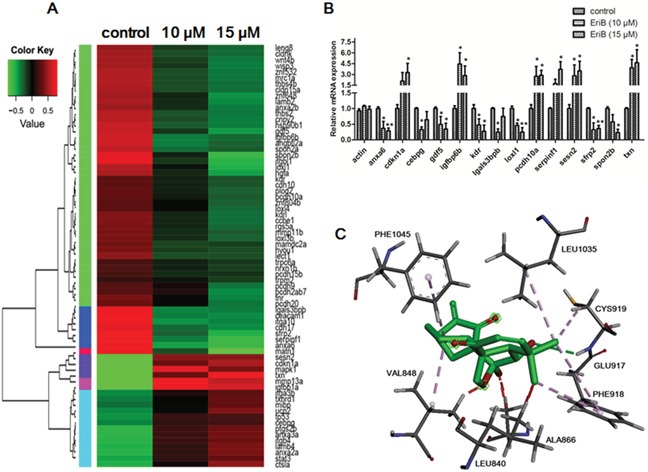
EriB exerted anti-angiogenic effect via the modulations of angiogenic genes expression and interaction with the ATP-binding sites of VEGFR-2 **A.** Heat map of 72 angiogenic genes (Supporting Information Table S1) expressions in zebrafish embryos after treatment with control or EriB (10 and 15 μM) for 72 h determined by transcriptome analysis (p < 0.05). The up-regulated mRNA expression in treated group with respect to control was represented by red colour and down-regulated mRNA expression was presented as green colour. The scale of color intensity was positively correlated to the fold change. **B.** Real-time PCR validation of selected gene expressions (Fold change >2). The specific genes name: *anxa6*, annexin A6; *cdkn1a*, cyclin-dependent kinase inhibitor 1A; *Cebpg*, CCAAT/enhancer binding protein (C/EBP), gamma; *gdf5*, growth differentiation factor 5; *igfbp6b*, insulin-like growth factor binding protein 6b; *kdr*, kinase insert domain receptor; *lgals3bpb*, galactoside-binding, soluble, 3 binding protein b; *loxl1*, lysyl oxidase-like 1; *pcdh10a*, protocadherin 10a; *serpinf1*, serpin peptidase inhibitor, clade F (alpha-2 antiplasmin, pigment epithelium derived factor), member 1; *sesn2*, sestrin 2; *sfrp2*, secreted frizzled-related protein 2; *spon2b*, extracellular matrix protein; *txn*, thioredoxin. **C.** The interactions of EriB to the amino acid residues in the ATP-binding site of VEGFR-2. EriB could stably bind to the ATP-binding pocket near the hinge region, with the interaction with residues Cys 919, Phe 1045, Val 848, Glu 917, Phe 918, Leu 1035, Leu 840.

In order to identify the underlying mechanisms that are responsible for the EriB regulated SIVs formation, transcriptome profiling were performed to compare EriB-treated zebrafish embryos (10 and 15 μM) to vehicle-treated embryos (0.1% DMSO) [27]. Results showed that the EriB treatment affected more than 1500 annotated genes, and a heat map of all the differentially expressed genes (DEGs) was generated (Supporting Information, Figure S1A). To further obtain relevant biological information, DEGs were used for functional analysis using Integrated Discovery (DAVID) software tools (david.abcc.ncifcrf.gov/home.jsp). Our findings revealed that EriB regulated a wide range of pathways, such as cell adhesion molecules (CAMs), ECM-receptor interaction, p53 signaling pathway, which were greatly associated with angiogenesis (Supporting Information, Figure S1B). Functional analysis of all the changed genes showed that cell adhesion, biological adhesion, cell growth, cell cycle arrest, cell motion, angiogenesis, blood vessel morphogenesis were the main biological processes in the regulation of angiogenesis (Supporting Information, Figure S1C). Multiple cellular components crucial for angiogenesis were also affected by EriB treatment, such as plasma membrane part, receptor complex, integrin complex, extracellular matrix, proteinaceous extracellular matrix (Supporting Information, Figure S1D). The vascular endothelial growth factor receptor activity was also significantly suppressed when treated with EriB from molecule function analysis (Supporting Information, Figure S1E). Additionally, protein-protein interaction prediction showed that vascular endothelial growth factor receptor (VEGF-R) was altered, which may play a vital role in the regulation of SIV formation (Supporting Information, Figure S1F). Figure 2A showed the identified 72 different angiogenic genes with 21 up-regulated and 51 down-regulated after treatment with vehicle control (0.1% DMSO) or EriB (10 and 15 μM) for 72 h using a cut-off point (p < 0.05) and method described in the materials and methods section [28]. Results showed that the expressions of significantly altered genes (Fold-change > 2) were consistent with the alterations from transcriptome profiling validated by Real-time PCR (Figure 2B).

### EriB binded with ATP-binding sites of VEGFR-2 kinase domain

The obtained information from DAVID suggested that EriB might interact with VEGFR leading to the inhibition of VEGFR activity. VEGFR-2, acting as the key regulator of angiogenesis, aroused our interests to implement further investigation. Next, molecular docking studies were applied to analyze the binding pattern between EriB and VEGFR-2 kinase domain. As shown in Figure 2C, EriB could stably bind at the ATP-binding site of VEGFR-2, and seven amino acids were actively involved (e.g. Cys 919, Phe 1045, Val 848, Glu 917, Phe 918, Leu 1035, Leu 840). On one side of EriB, hydrogen bonds were formed with the residues Cys 919 and Phe 1045, accompanying with following favorable interaction between EriB and Leu 1035, Leu 840 of VEGFR-2. Our findings indicated that the interactions of EriB to VEGFR-2 resulted in the competitive inhibition to ATP against VEGFR-2, leading to the prevention of VEGFR-2 phosphorylation with a docking score of 21.19 kJ/mol evaluated by Chemscore.

### EriB inhibited VEGF-induced cell viability and cell proliferation in HUVECs

**Figure 3:**
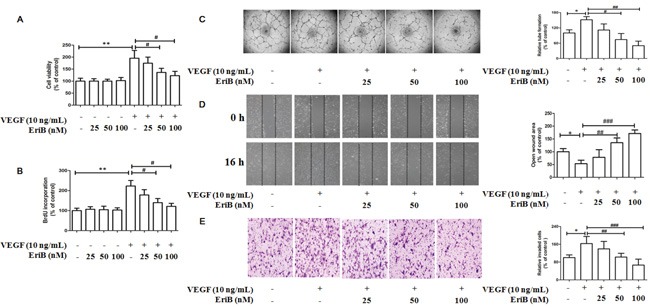
EriB inhibited VEGF-induced cell viability, cell proliferation and also suppressed *in vitro* angiogenesis in HUVECs **A.** Different concentrations of EriB (25, 50 and 100 nM) were added to HUVECs in the absence or presence of VEGF (10 ng/mL) for 48 h, then the cell viability were assessed by MTT assay. **B.** BrdU incorporation assay was performed to detect the effect of EriB on cell proliferation when treated with EriB for 48 h in the absence or in the presence of VEGF. **C.** EriB inhibited the VEGF-induced tube formation after incubation for 8 h and tube structures of HUVECs were photographed (magnification, ×40). **D.** EriB suppressed the VEGF-induced cell migration after the incubation for 16 h and the wounded area of each well was captured at 0 h and 16 h of incubation (magnification, ×40). **E.** EriB attenuated VEGF-induced cell invasion after 8 h incubation, the migrated cells on the lower side of membranes were stained and counted (magnification, ×100). Each value was presented as means + SD (n=3). * p < 0.05, ** p < 0.05 compared with control, ^#^ p < 0.05, ^##^ p < 0.01, ^###^ p < 0.001 compared with VEGF alone group (one-way ANOVA).

To investigate the *in vitro* anti-angiogenic activity, the inhibitory effects of EriB on cell viability and cell proliferation in the absence or presence of VEGF were evaluated. HUVECs were subjected to different concentrations of EriB (25 nM, 50 nM, 100 nM) and VEGF (10 ng/mL) before MTT assay. As shown in Figure 3A, cell viability was significantly increased in the presence of VEGF for 48 h. However, when treated with different non-toxic concentrations of EriB, cell viability was obviously reduced in a dose-dependent manner (Supporting Information, Figure S4A & S4B).

To clarify whether the decreased HUVECs viability was associated with the inhibition of cell proliferation, BrdU labelling analysis was performed. A significant increment of cell proliferation was also observed when incubated with VEGF for 48 h, which was reduced by EriB treatment in a concentration-dependent manner (Figure 3B). Additionally, exposure of HUVECs in the absence of VEGF failed to inhibit cell viability and cell proliferation, suggesting that EriB had greater specificity as an inhibitor for VEGF-induced endothelial compared to the unstimulated group.

### EriB inhibited VEGF-induced tube formation, cell migration and cell invasion

During the blood vessel sprouting, tube formation, endothelial cells migration and invasion are necessary steps in angiogenesis process. Thus, *in vitro* angiogenesis models were used, such as tube formation assay, cell migration assay and cell invasion assay.

Regarding the effects of EriB on tube formation, approximately 40 % and 60 % tube formation was inhibited when treated with EriB at 50 nM and 100 nM for 11 h, respectively (Supporting Information, Figure S2A). Moreover, VEGF treatment obviously induced tube formation after incubation for 8 h, leading to a significant reduction of tube number, length, and area (Figure 3C) in a dose-dependent manner. The effects of EriB on HUVEC migration were evaluated using scratch wound assay. It was found that EriB (50 and 100 nM) could significantly inhibit cell migration after 24 h (Supporting Information, Figure S2B). Furthermore, a strong inhibitory effect of EriB on VEGF-induced tube formation was also observed when treated for 16 h (Figure 3D) dose-dependently. Transwell invasion assays were performed to determine the invasion activities of HUVECs passing through the membrane to the lower layer. The cell invasion ability was reduced by EriB (50 and 100 nM) treatment compared with control group (Supporting Information, Figure S2C). As shown in Figure 3E, an obvious inhibitory activity of EriB on VEGF-induced endothelial cell invasion was observed.

### EriB caused G1 arrest via the modulation of p21-cyclin D1/CDK4-pRb pathway

**Figure 4:**
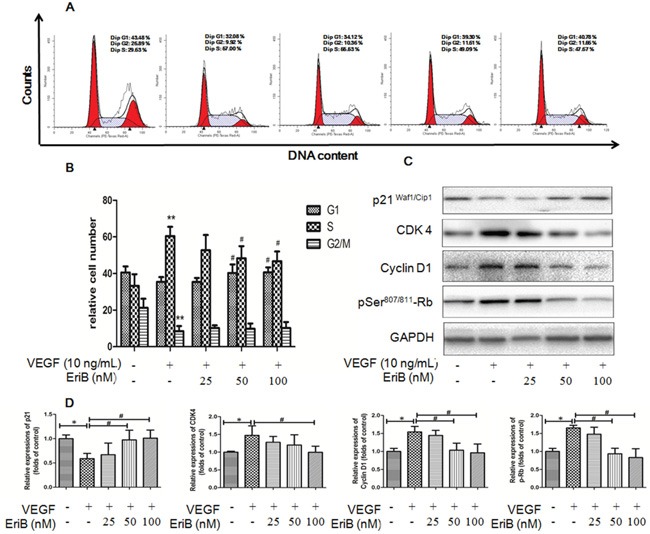
EriB caused G1 arrest via the modulation of p21-cyclin D1/CDK4-pRb pathway **A.** Representative histograms of the cell cycle obtained after flow cytometry analysis when HUVECs were treated with EriB (25, 50 and 100 nM) in the presence of VEGF (10 ng/mL) for 24 h. **B.** Quantification of the cell populations in each phase of the cell cycle was presented as means + SD (n=3). ** p < 0.01 compared with control, ^#^ p < 0.05 compared with VEGF group (one-way ANOVA). **C.** HUVECs were incubated with EriB and VEGF (10 ng/ml) for 16 h. Then the cells were harvested and western blotting was performed. **D.** The histograms showed quantified results of protein levels, which were adjusted with corresponding GAPDH protein level. Each value was expressed as fold of control mean + S.D. (n=3). * p < 0.05 compare with control, ^#^ p <0.05 compared with VEGF group (one-way ANOVA).

Our results revealed that EriB treatment without any cytotoxicity exhibited anti-proliferative effect in HUVECs, so we wonder the association between the cell proliferation and cell cycle progression. Flow cytometry of HUVECs was performed after the treatment with VEGF for 6 h to 48 h. The percentage of cells in S phase was obviously elevated during the period of time from 12 h to 24 h and dropped significantly afterwards (data not shown). Figure 4A demonstrated that VEGF induced cell types into S phase, whereas addition of EriB markedly reduced S phase entry at 24 h, leading to the increased proportion of cells in G0/G1 phase and a corresponding decrease in S phase in a dose-dependent manner (Figure 4B). Thus, these results indicated that EriB could affect the transition of cells from G1 phase to S phase.

To critically explain the involvement of cyclins and cyclin-dependent kinases during G1 phase arrest, western blot was applied. Figure 4C & 4D showed that VEGF treatment enhanced cyclin D1, CDK4, pRb levels with a decrease in p21 (WAF1/Cip1) expressions at 16 h. However, these effects were significantly reversed by EriB treatment, suggesting that EriB was most likely to interfere with this p21-cyclin D1/CDK4-pRb pathway.

### EriB inhibited activation of VEGFR-2 induced by VEGF and suppressed VEGFR-2-mediated downstream signaling pathway

Accumulating evidences had suggested the VEGFR-2 and VEGFR-2-mediated downstream signaling pathway play a critical role in physiologic and pathologic angiogenesis regulation [12, 13]. Since our results had already indicated the interaction between EriB and VEGFR-2, it was reasonable for us to test whether EriB exhibited its anti-angiogenic effect through the modulation of VEGFR-2 and its downstream signaling pathways in HUVECs.

**Figure 5:**
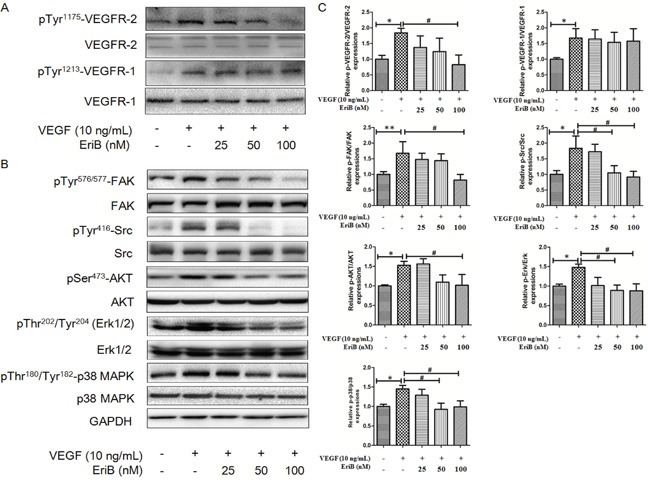
EriB inhibited the activation of VEGFR-2 induced by VEGF and suppressed VEGFR-2-mediated downstream signaling pathway **A.** HUVECs were pretreated with various concentrations (25, 50 and 100 nM) of EriB for 24 h before exposure to VEGF (10 ng/mL) for 30 min. Then the cells were harvested and western blotting was performed to detect pTyr^1175^-VEGFR-2 pTyr^1213^-VEGFR-1 and total VEGFR-2, VEGFR-1 expressions. **B.** HUVECs were pretreated for 24 h with various concentrations (25, 50 and 100 nM) of EriB before exposure to VEGF (10 ng/mL) for 120 min. Then whole cell extracts were extracted for western blotting analysis to detect modulation of VEGFR-2-mediated signaling pathway. **C.** The histograms showed quantified results of protein levels, which were adjusted with corresponding GAPDH protein level. Each value was expressed as fold of control mean + S.D. (n=3). * p < 0.05, ** p < 0.01 compared with control, ^#^ p < 0.05 compared with VEGF group (one-way ANOVA).

As shown in Figure 5A, the addition of VEGF resulted in the induction of VEGFR-2 phosphorylation in 30 min [10, 29]. However, VEGFR-2 activation was specifically inhibited without affecting the overall VEGFR-2 levels when pretreated with EriB (100 nM) for 24 h. Furthermore, the levels of phosphorylated and total forms of VEGFR-1 were not affected by EriB, indicating that only VEGFR-2 but not VEGFR-1 was involved in this process. This result was consistent with molecular docking study, indicating that EriB might enter the ATP-binding pocket of VEGFR-2 kinase domain and exhibited comparatively greater binding affinity, subsequently prevented VEGFR-2 phosphorylation.

We further detected several downstream pathways involved in the process of VEGFR-2-mediated angiogenesis. The levels of phosphorylated steroid receptor coactivator (Src) and focal adhesion kinase (FAK) were enhanced by VEGF treatment without any alteration on total protein expressions. Figure 5B & 5C showed that the phosphorylation kinases levels of Akt, Erk, Erk1/2 and p38 MAPK were significantly decreased without affecting total levels after EriB treatment. All these findings suggested that EriB exerted its anti-angiogenic function through the blockage of VEGFR-2-mediated signal transduction (Supporting Information, Figure S4F & S4G).

### EriB exerted anti-angiogenic effects of in mouse matrigel plug model

**Figure 6:**
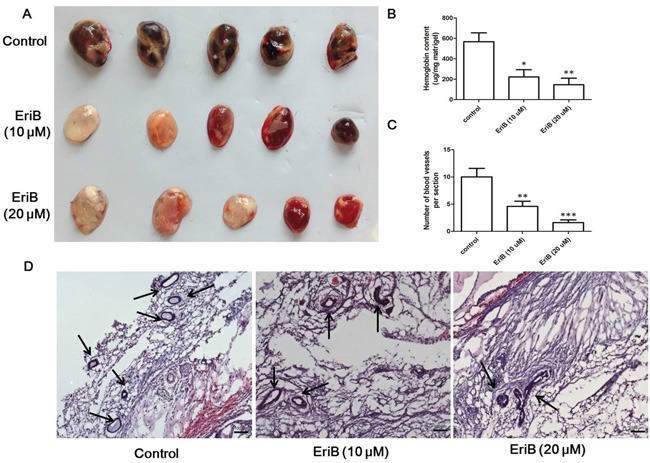
EriB-mediated anti-angiogenesis effect in Matrigel plug assay **A.** C57 mice were injected with Matrigel in the presence or absence of EriB (10 and 20 μM) for 7 days and sacrificed to obtain the Matrigel plugs. The appearance of Matrigel plugs from each group was presented. **B.** Hemoglobin content of Matrigel plugs from each group was measured using Drabkin's reagent kit. Each value was presented as means + SEM (n=3). * p < 0.05, ** p < 0.01 compared with control (one-way ANOVA). **C.** Quantification of the number of blood vessels in frozen sections of the Matrigel plugs were presented as means + SD (n=2). ** p < 0.01, *** p < 0.001 compared with control (one-way ANOVA). **D.** Representative photos of blood vessels stained with H&E (magnification, ×100), scale bar: 100 μm.

To further investigate the anti-angiogenic effect of EriB *in vivo*, the Matrigel plug assay was performed. The plugs in red color containing bFGF and heparin abundantly filled with intact red blood cells (RBCs) indicated the formation of many new blood vessels inside the Matrigel. As shown in Figure 6A, the red color of plugs were much lighter or even looks pink in the presence of EriB compared with the control group, suggesting the formation of fewer blood vessels. In Figure 6B, histogram showed that the hemoglobin concentrations in the plugs loaded with EriB were significantly lowered than those plugs without EriB, indicating that EriB exerted anti-angiogenic effect *in vivo*. Notably, the number of blood vessel formation induced by bFGF was markedly reduced by EriB from the H&E staining (Figure 6C, 6D and Supporting Information, Figure S4E).

### EriB inhibited tumor angiogenesis and suppressed tumor growth in mouse 4T1 breast tumor model

**Figure 7:**
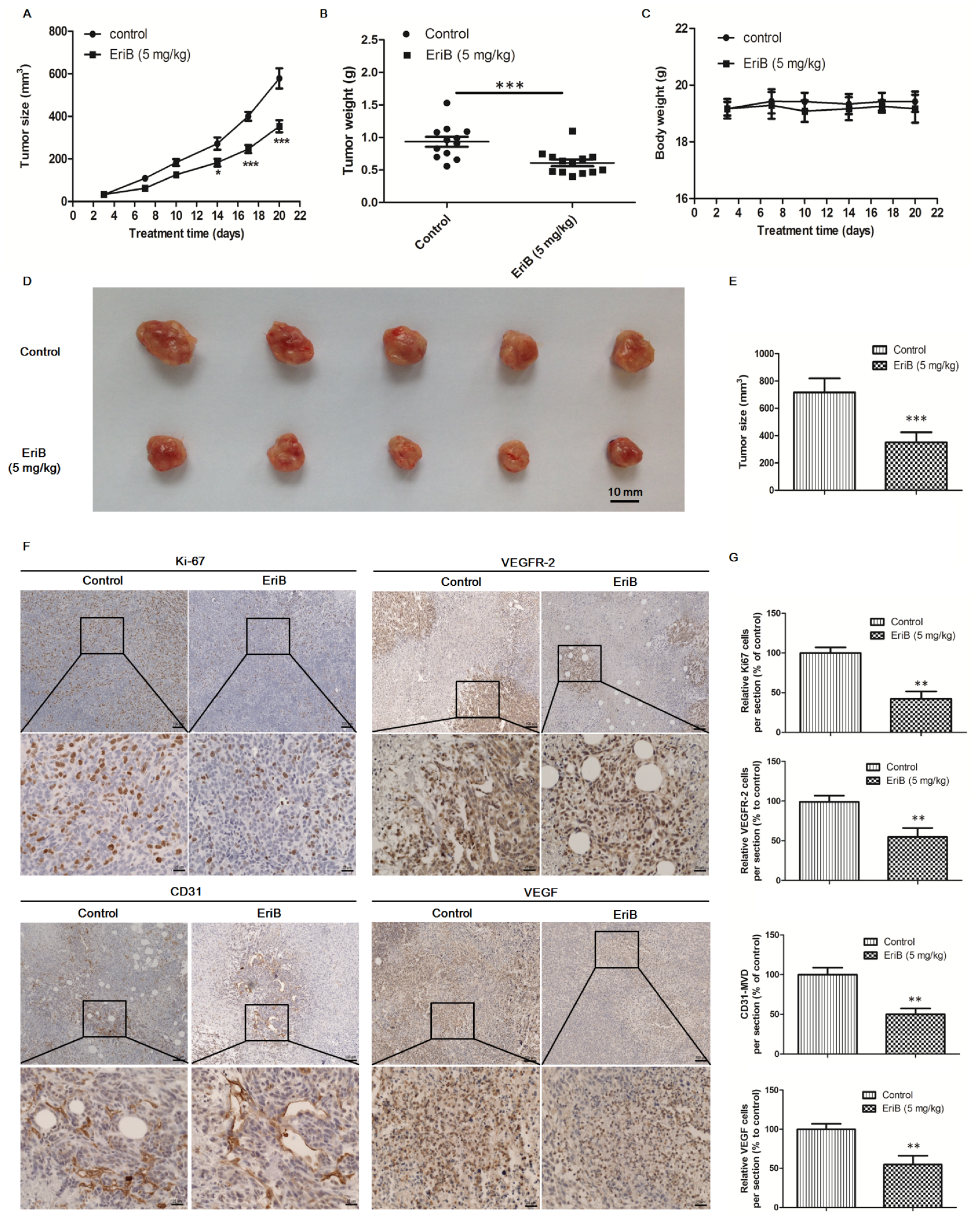
EriB inhibited tumor angiogenesis and suppressed tumor growth in mouse 4T1 breast tumor model Female BALB/c mice were injected subcutaneously with 4T1 cells for 6 days before EriB treatment **A.** The growth of 4T1 xenograft tumors in mice after control (vehicle), EriB (5 mg/kg) treatments. The final tumor weight **B.** and body weights **C.** were measured. Each value was presented as means + SEM (n=13). * p < 0.05, *** p < 0.001 compared with control (Student's t-test). Representative tumor image **D.** and tumor size **E.** was shown. **F.** 4T1 tumor tissues were stained with anti-Ki67 (brown staining), anti-VEGFR-2 (brown staining) anti-VEGF (brown staining), and anti-CD 31(blood vessels) antibodies (magnification, ×100, ×400), scale bar: 100 μm, 25 μm. **G.** Results are shown as the relative percentages of the positive cells in the total number of cells per section. Each value was presented as means + SD (n=4). ** p < 0.01, *** p < 0.001, compared with control (Student's t-test).

Tumor angiogenesis supplies oxygen and nutrients to maintain tumor growth, invasion and metastasis, which is essential for tumor progression [5]. To further determine the inhibitory effect of EriB on tumor angiogenesis and tumor growth, a mouse 4T1 breast tumor model was employed (Supporting Information, Figure S4C). As shown in Figure 7A, tumor sizes in control group were rapidly increased after 21 days, whereas in EriB treated group, it increased much slower. Also the final weight of tumors in EriB-treated group was different from control group (p < 0.001, Figure 7B). There was no obvious body weight loss (Figure 7C) and no alteration of the plasma enzyme activity (Supporting Information, Figure S3) was observed in EriB-treated group, suggesting that EriB could inhibit tumor growth (Figure 7D & 7E) in xenograft mouse breast tumor model without any toxicity. Immunohistochemical results of Ki67 (proliferative index), VEGFR-2 (proliferative index, grade of malignancy index), VEGF (proliferative index, grade of malignancy index) [30] revealed that EriB treatment inhibited cell proliferation and cell growth in solid tumors (Figure 7F, 7G and Supporting Information, Figure S4D). Furthermore, microvessel density (MVD) quantified by CD31-stained cells was significantly decreased (Figure 7G) through the hotspot method by imaging high power fields [31, 32].

## DISCUSSION

Eriocalyxin B (EriB), a natural compound isolated from *I*. *eriocalyx* var. *laxiflora*, which possesses multiple anti-tumor and anti-inflammation activities [20–24]. However, little is known about the inhibitory activities of EriB on angiogenesis and the underlying mechanisms. In the present study, our findings provided evidences that EriB has anti-angiogenic activity both *in vitro* and *in vivo*. It exerted significant inhibitory effect on HUVECs' function by decreasing endothelial cell viability, proliferation, suppressing tube formation, cell migration and cell invasion *in vitro*. Furthermore, different animal models were applied in our present study to evaluate the anti-angiogenic effect of EriB. *In vivo* results showed that EriB treatment at non-toxic dose significantly suppressed the formation of subintestinal vessels (SIVs) in zebrafish embryos, blocked angiogenesis in Matrigel plug model and inhibited the tumor growth via the suppression of tumor angiogenesis in mouse breast tumor model.

Zebrafish embryo is an efficient screening system used for high-throughput screening of compounds which affect angiogenesis, as the blood vessel can be stained and visualized microscopically [33]. In the present study, it was shown that the inhibition of subintestinal vessels (SIVs) formation by EriB in zebrafish embryos was correlated with the alteration of angiogenic genes expressions from transcriptome analysis. For example, the up-regulation of *cdkn1a* (p21) was observed which is vital in the regulation of cell cycle, as well as the elevated expression of *serpin f1* (Pigment epithelium-derived factor, PEDF), which is a strong anti-angiogenic gene [34]. Additionally, GO functional analysis and KEGG pathway enrichment analysis also demonstrated that EriB treatment could modulate several biological processes, such as cell-cell and cell-matrix adhesion, cell growth, cell cycle arrest and blood vessel morphogenesis, all of which may be regulated by cell adhesion molecules (CAMs), ECM-receptor interaction, and p53 signaling pathways.

Protein-protein interactions (PPIs) prediction revealed the involvement of VEGFR as well as the reduction of VEGFR activity from molecule function analysis. Interestingly, the down-regulated *kdr* (VEGFR-2) mRNA level was observed, which is a prominent regulator in VEGF signaling pathway regulating vasculogenesis and angiogenesis in zebrafish development [35–37]. Additionally, both quiescent and activated VEGFR-2 can be constitutively recycled back to the plasma membrane or undergoes degradation within the endosome-lysosome system [38]. In addition, plasma membrane part, VEGFR complex and integrin complex were also controlled by EriB. Thus, EriB treatment in zebrafish embryos may probably interfere with *kdr* trafficking, proteolysis and signaling status as well as endosome-to-plasma membrane recycling processes, which were predicted to suppress *kdr* signaling and reduce cell surface *kdr* levels during the formation of subintestinal vessels (SIVs).

To further evaluate the interaction between EriB and VEGFR-2, docking analysis was performed. Structurally, VEGFR-2 is composed of 1356 amino acids which can be divided into the N-terminal lobe and the larger C-terminal lobe [11]. ATP-binding site of VEGFR-2 tyrosine kinase is located between the N- and C-terminal lobes of the catalytic domain, which is essential for the activation of VEGFR-2 kinase activity. Previous studies demonstrated that a variety of natural angiogenesis inhibitors, such as gamabufotalin [17], ellagic acid [18], tryptanthrin [19] prevented VEGFR-2 phosphorylation via competing with cellular ATP binding to the ATP-binding sites. The present study revealed that EriB could stably bind to the ATP-binding pocket of VEGFR-2 through a favorable interaction among seven amino acids (e.g. Cys 919, Phe 1045, Val 848, Glu 917, Phe 918, Leu 1035, Leu 840), which further supported our hypothesis that VEGFR-2 was involved in the EriB-exerted anti-angiogenic effect.

HUVECs, the most commonly used endothelial cells for angiogenesis research were chosen in the present investigation. VEGF is a specific mitogen and survival factor essential for endothelial cells *in vitro*, which is also a potently angiogenic *in vivo*. Here, we reported that EriB at non-toxic concentrations inhibited VEGF-stimulated cell viability and cell proliferation. Treatment of HUVECs with EriB effectively inhibited tube formation, cell migration and cell invasion (Supporting Information, Figure S2). Furthermore, a greater inhibitory effect was observed in VEGF-induced angiogenesis *in vitro*, which may be due to alteration of biological activities of EriB as well as the enhancement of the membrane permeability, and solubility by VEGF [39]. Previous studies had reported that the inhibition of cell cycle progression is capable to suppress endothelial cell proliferation and block angiogenesis [40].

Flow cytometry results demonstrated that VEGF stimulated cell cycle progression from G1 to S phase, which was result from the induction of Rb phosphorylation and the corresponding up-regulation of cyclin D1 and Cdk4 kinase expressions [41]. However, the transition of cells from G1 to S was suppressed by EriB. To further elucidate the underlying mechanism of the cell cycle arrest induced by EriB, the relevant proteins expressions were determined. From our western blot data, the up-regulation of the hyper-phosphorylation of pRb was observed as early as 18 h (data not shown), which was inhibited when treated with EriB. In addition, EriB was also able to inhibit VEGF-induced cyclin D1 and CDK4 expressions and increase the Cdk inhibitor p21Waf1 levels. Thus, our findings suggested that EriB may most likely interfere with this p21-cyclin D1/CDK4-pRb pathway and inhibit DNA synthesis and cell cycle progression consequently [42, 43].

It is widely accepted that VEGFR-1 plays a dual role in the regulation of angiogenesis while VEGFR-2 appears to be the dominant mediator in VEGF-driven responses in endothelial cells. Binding of VEGF to VEGFR-2 contributes to the activation of VEGFR-2 signaling, which regulates cell proliferation, migration, differentiation, capillary like formation and vascular permeability [7]. Our results showed that EriB (100 nM) inhibited VEGF-induced VEGFR-2 phosphorylation but not VEGFR-1 in HUVECs, indicating that VEGFR-2 is the major regulator responsible for the modulation of angiogenesis by EriB, which was consistent with the docking result. Previous studies have suggested that the phosphorylation of VEGFR-2 is an ATP-consuming process [17, 44, 45]. The underlying mechanisms of the inhibitory effects on VEGFR-2 kinase activity may probably due to the entry of EriB into ATP-binding pocket of VEGFR-2 domain, exerting a favorable interaction among seven amino acids, leading to the enhancement of the conformational stability of the EriB/VEGFR-2 complex and reduction of VEGFR-2 phosphorylation levels consequently.

We further examined whether EriB was capable to block the downstream events of VEGFR-2 signaling kinases, such as FAK, Src, Akt, ERK, p38 MAPK and JNK, etc. It has been reported that FAK is activated by phosphorylated VEGFR-2 and then acted as a substrate for steroid receptor coactivator (Src) to form a dual FAK-Src kinase complex [46, 47]. This process has the potential to phosphorylate other substrates and trigger multiple intracellular signaling pathways, including PI3K/PTEN/Akt/mTOR, Ras/Raf/MEK/ERK [48], p38 MAPK and SAPK/JNK signaling cascades [7], all of which play a critical role in cell proliferation, survival, migration and adhesion. After treated with EriB in the presence of VEGF, phosphorylated Akt and ERK were decreased apparently, which appears to be correlated with cell survival and proliferation, respectively. In addition, activation of p38 MAPK is a necessary requirement for VEGF-stimulated proliferation and migration, which was also attenuated by EriB. The anti-angiogenic activity of EriB *in vivo* was further determined using Matrigel plug assay. EriB remarkably suppressed sprouting of endothelial cells, reduced new blood formation in Matrigel plug. Thus, both *in vitro* and *in vivo* results provided important evidences that EriB is an anti-angiogenic agent. Nonetheless, it will be interesting to explore the potential synergistic interaction between EriB and some well-known anti-angiogenic drugs (e.g. bevacizumab). The possibilities of EriB increasing the efficacy of anti-angiogenic agents are worth to be further explored in the future.

Moreover, angiogenesis is essential for tumor growth, which supplies nutrients and oxygen in tumor cell intravasation and dissemination escaped from the primary tumor sites. Thus, the anti-tumor activity of EriB was evaluated using a mouse 4T1 breast tumor model. It is notable that EriB could significantly suppress tumor growth without any change on body weights and no alteration of the plasma enzymes activity (Supporting Information, Figure S3). It further supported the previous animal study showing that EriB has less side effect. Immunohistochemical analysis of Ki67, VEGFR-2, VEGF and CD31 also revealed that EriB treatment inhibited tumor cells proliferation, tumor angiogenesis, which may partly explain the anti-tumor activity of EriB *in vivo*.

Taken together, our findings clearly demonstrated that EriB exhibited significant anti-angiogenesis activity through the modulation of the VEGFR-2 mediated signaling pathway both *in vitro* and *in vivo*. It may serve as a potent inhibitor of VEGFR-2 stimulated angiogenesis and could be developed into a potential candidate for treatment of angiogenesis-dependent human diseases, such as cancers.

## MATERIALS AND METHODS

### Chemicals and reagents

EriB (Figure 1A) was isolated from *I*. *eriocalyx* var. *laxiflora* with purity over 95% as previously described [23]. EriB in powder form was dissolved in DMSO at a concentration of 100 mM and stored at −20°C. Phospho-VEGFR1 (Tyr^1213^) was from R&D (MN, USA). VEGFR1, VEGFR-2, Phospho-VEGF Receptor 2 (Tyr^1175^), Phospho-Akt (Ser^473^), Akt, Phospho-Src Family (Tyr^416^), Src, Phospho-FAK (Tyr^576/577^), FAK, Phospho-p38 MAPK (Thr^180^/Tyr^182^), p38 MAPK, Phospho-p44/42 MAPK (Erk1/2) (Thr^202^/Tyr^204^), Erk1/2, CDK2, CDK6, Cyclin E, Cyclin D1, Cyclin A, p21 (Waf1/Cip1), CDK4, Phospho-Rb (Ser^807/811^), and GAPDH antibodies were all obtained from Cell Signaling Technology (MA, USA). β-actin was from Sigma (MO, USA). Basement membrane matrix Matrigel (Growth factor reduced) was from BD Biosciences (CA, USA). RNeasy Mini Kit for RNA isolation and QuantiFast SYBR Green RT-PCR Kit for Real-time PCR were obtained from QIAGEN (Martinsried, Germany).

### Maintenance of zebrafish and collection of embryos

The transgenic zebrafish line Tg(*fli1*:EGFP)y1 was obtained from Zebrafish International Resource Center, University of Oregon (USA). In the experiment, healthy embryos were collected and treated with different concentrations of EriB for 72 h. Photos of the subintestinal vessels (SIVs) were taken and analyzed as described in our previous study [14].

### Transcriptome profiling of EriB treated zebrafish

After the confirmation of inhibition on SIVs formation under the microscope, total RNA of the whole zebrafish embryo was extracted after EriB treatment for 72 h as previously described [27]. Paired-end transcriptome sequencing was performed at the Beijing Genome Institute (BGI-Shenzhen). The zebrafish genome sequence and annotation (GRCz10) were downloaded under the accession number GCF_000002035.5 in the Ref Seq database. The reads were mapped on the reference genome by Tophat, followed by Cufflinks to analyze the expression level of each gene and identify the differentially expressed genes [49]. The p-value less than 0.05 was regarded as statistically significant. The differentially expressed genes were analyzed in the Database for Annotation, Visualization and Integrated Discovery (DAVID), which could identify the Gene Ontology (GO) terms and KEGG pathway maps. The angiogenesis related genes were identified according to GO enrichment analysis and Angiogenic Growth Factors RT^2^ Profiler PCR Array (SABiosciences, Frederick, MD, USA) [28].

### Real time-PCR analysis

Total RNA of the whole zebrafish embryo was extracted using Trizo1 reagent to further confirm the alteration of angiogenic genes expressions. The primers sequences were listed in Table 1. The reverse transcription and the quantification were performed according to our protocol described before [14].

**Table 1:** Gene specific PCR primers

Gene name	Forward primer	Reverse primer
*actin*	TCCCCTTGTTCACAATAACC	TCTGTTGGCTTTGGGATTC
*anxa6*	AAATGTGGGAGATCAGCGCC	AATATGGCCGCACAGTTCCT
*cdkn1a*	GGAGCTGCATTCGTCTCGTA	AACGGTGTCGTCTCTGGTTC
*cebpg*	AACCACAGAAAGTCCGACCC	CCCTCCAAGTCAGCACAAGC
*gdf5*	CTCAGGTAAAAACGCAGCCG	TGAGAGTCGAAAGGTCTCGC
*igfbp6b*	AAACTGTGACACTCGTGGCT	CCCTTTGAGGACCGACACTG
*kdr*	AGGACCCAGACTATGTCCGCAAAG	GGATGTAGTGCTTTCCCTCCTG
*lgals3bpb*	TCAGTTCAACGCTCTTCCCTT	AGTGCTCACATTCAAACCGAG
*loxl1*	GTTTGGATTCGGACAGGTGC	GAGAGCCGGTTTGTGACTGA
*pcdh10a*	TACGCCATCACCACGAACAA	GCACGTATTTGTGGATCGCC
*serpinf1*	ATGCTCAGTTGGCAGACACA	TGACGGAACAGGTTGTAGC
*sesn2*	TCCTTCCCGATTGTGTGTCG	GCCGTGATCCTCCCATGAAT
*sfrp2*	ACGAATCCTTCATTTAGCGTCA	AAACAAAGCTGGAAGCGCAG
*spon2b*	TGCTCAGTGGTCCCCTCTTA	GTCCAAGCCTCTGCTCTCTC
*txn*	ACAGGATGTGGCCGCTTTAT	CTCCTCCAGCTTGGATTGGTT

### Molecular docking

In order to further understand how EriB inhibited the activation of VEGFR-2 kinase and its downstream signaling pathways, docking analysis was performed to detect the direct interactions of EriB with VEGFR-2 tyrosine kinase. Discovery Studio 3.5 (Accelrys) and GOLD [50] were used for molecular docking studies [26]. The crystal structure of VEGFR-2 tyrosine kinase obtained from Protein Data Bank (PDB entry: 3VID and 2OH4) (http://www.rcsb.org/pdb/), which contains a phosphorylated activation loop, was used for the docking study [51]. The structure 3VID was selected as an EriB similar small molecule ligand co-crystalized. However, some residues missed in 3VID, thus 2OH4 was then used as a template to complete the structure of 3VID through homology modelling. The ligand in 3VID was used as the reference to define the ligand-binding site during docking study [52]. In the docking process, EriB was docked into VEGFR-2 using default parameter setting with the ChemScore fitness function and the best-fitting binding mode was identified as the output for a particular ligand.

### Cell culture and treatment

Human umbilical vein endothelial cells (HUVECs) were obtained from Lonza (Walkersville, MD, USA) and maintained in passages 3-8 to ensure genetic stability. Recombinant vascular endothelial growth factor (VEGF) and heparin were obtained from Sigma (MO, USA); endothelial cell growth supplement (ECGS) was from Upstate (Biotechnology, USA); fetal bovine serum and penicillin–streptomycin were from Invitrogen (CA, USA). HUVECs were cultured in M199 medium supplemented with heparin (90 mg/ml), heat-inactivated FBS (20%, v/v), ECGS (20 mg/ml), and penicillin/steptomycin (PS, 1%, v/v) and kept at 37°C in a humidified 5% CO_2_ incubator. All the assays in HUVECs from passages 3 to 8 were starved in 1% FBS supplemented M199 medium for 16 h and then incubated with or without VEGF and EriB for the indicated period.

### Cytotoxicity assay and cell proliferation assay

HUVECs (3×10^3^ cells/well) were seeded onto 96-well plates overnight and then cells were treated with various concentrations of EriB in the presence or absence of VEGF (10 ng/mL) for 48 h. Cell viability and cell proliferation were determined according to the procedures described previously [24].

### Tube formation assay

The 96-well plate pre-coated with 60 μL growth factor reduced Matrigel was allowed to solidify at 37°C for at least 30 min, which was essential for HUVECs to form capillary-like structure. HUVECs (2×10^4^ cells/well) were seeded in 1% FBS supplemented medium containing different concentrations of EriB in the presence or absence of VEGF (10 ng/mL) for 8 h or 11 h. The procedures were according to the procedure described previously [14].

### Cell migration assay

HUVECs (3×10^4^ cells/well) were seeded onto 96-well plates for 24 h. An artificial wound was created by mechanical scratching of the cell monolayer using pipette tips and washed with PBS after starvation for 16 h. The sharp wound image in each well was captured. Culture medium was replaced with fresh medium treated with various concentrations of EriB in the presence or absence of VEGF (10 ng/mL) for another 16 h or 24 h. The migrated cells of each well were captured under a microscope again. Images taken at different time points were analyzed using ImageJ software.

### Transwell invasion assay

Invasion assay was performed using a transwell (Corning, NY, USA) as described previously [14]. Briefly, the lower chambers were filled with M199 medium containing 20 % FBS after coating with 0.1 % gelatin. The upper chambers were seeded with HUVECs in the presence or absence of VEGF (10 ng/mL) together with different concentrations of EriB in M199 medium containing 1 % FBS. Cells were allowed to migrate for 8 h or 11 h. Non-migrated cells were scraped with a cotton swab, and migrated cells were fixed and stained with 0.5% toluidine blue in 4% paraformaldehyde. The cells were photographed under a microscope (Olympus IX-71) and quantified by counting the number of stained cells in three random fields.

### Cell cycle analysis

HUVECs (1×10^5^ cells/well) were seeded in 6-well culture plates and incubated overnight to allow attachment. Different concentrations of EriB in the presence or absence of VEGF (10 ng/mL) were added to the wells and incubated for 24 h after starvation. At least 10,000 cells per sample were collected and analyzed by flow cytometry (CA, USA). Data were calculated using Modfit LT (ME, USA) as described previously [24].

### Western blot analysis

HUVECs (1×10^6^ cells/well) were seeded in 100 mm culture dish and incubated overnight to allow attachment. Various concentrations of EriB were added to the dishes and incubated for 24 h after starvation. After treatment with VEGF (10 ng/mL) for 30 min or 120 min, cells were lysed in lysis buffer (Biyuntian, China) and subjected to 8 % or 10 % SDS-PAGE according to the protocol described previously [14].

### *In vivo* matrigel plug assay

Female C57BL/6 mice (6 weeks old) were supplied and maintained by Laboratory Animal Service Center, the Chinese University of Hong Kong. Matrigel (400 μL) was mixed with heparin (500 U/mL), bFGF (500 ng/mL) in the presence or absence of EriB (10 μM, 20 μM), then subcutaneously injected into the flanks of C57BL/6 mice. After 7 days, the intact Matrigel plugs were removed and photographed. Half of the plugs were suspended in PBS to further quantify. The hemoglobin content in the matrigel plugs was determined using Drabkin's reagent kit (Sigma, USA). The other matrigel plugs were fixed with 4% PFA formalin for frozen sections (10 μm). Afterwards, H&E staining was performed to identify the formation and infiltration of new microvessels (magnification, 200X). Functional microvessels with red blood cells (RBCs) were counted manually.

### Mouse 4T1 breast tumor model

Female BALB/c mice (6–8 weeks old) were supplied and maintained by Laboratory Animal Service Center, the Chinese University of Hong Kong. The 4T1 breast tumor cells (5×10^5^ cells resuspended in 100 μL PBS) were subcutaneously inoculated at the mammary fat pad of BALB/c mice. After 4T1 cell inoculation for 6 days, the mice were randomly assigned into 2 groups: vehicle control group, and EriB group (5 mg/kg/day). EriB was dissolved in 1% Pluronic F-68 in saline and the intraperitoneal administration was initiated at the seventh day after inoculation for 21 consecutive days. During EriB treatment, the tumor size, body weight of each mouse was measured. At the end of experiment, whole blood was obtained from the mice by cardiac puncture after anesthesia. Plasma was collected and analyzed using an enzyme kits (Stanbio Laboratory, USA) according to previously reported method [24]. Tumors of mice from different groups were removed for histological analysis.

### Histological and immunohistochemical analysis

Tumors were fixed in 10% buffered formalin and embedded in paraffin. 5 μm tumor sections were stained with specific antibodies including CD 31 (Dianova, Germany), Ki-67 (Abcam, USA), VEGF (Abcam, USA), VEGFR-2 (Cell Signaling Technology, USA) as described previously [14]. Immunoreactive species were detected using 3, 3-diaminobenzidine tetrahydrochloride (DAB) as a substrate. Images were taken using an Olympus BX51 microscope and quantification of positive immunostaining cells in tumor sections was counted manually in a double-blind manner as described in our previous study [53]. Microvessel density (MVD) is quantified by CD31-stained cells via imaging high power fields (magnification, ×400) through hotspot method and calculated as the relative percentage of CD31 positive vessels in 4 fields of view according to the previous reports [31, 32].

All animal experiments in zebrafish and mice were carried out according to the approved guidelines specified by the Animal Experimentation Ethics Committee of the Chinese University of Hong Kong (CUHK). All experimental protocols were approved by the Animal Experimentation Ethics Committee of CUHK with reference numbers 10/013/MIS and 10/051/MIS [14].

### Statistical analysis

GraphPad prism 5.0 was used for statistical analysis. *In vitro* data were expressed as the mean + S.D. and *in vivo* data were summarized as mean + SEM or mean + S.D. One way analysis of variance (ANOVA) was used to determine the significant differences among the different groups *in vitro*, and Student's *t*-test was used for *in vivo* data analysis. p < 0.05 was considered as statistically significant.

## SUPPLEMENTARY FIGURES AND TABLES






