# Supplementary material for: Eriocalyxin B, a natural diterpenoid, inhibited VEGF-induced angiogenesis and diminished angiogenesis-dependent breast tumor growth by suppressing VEGFR-2 signaling
Source: Oncotarget. 2016 Oct 14;7(50):82820–35. doi: 10.18632/oncotarget.12652 (PMC5347735; doi:10.18632/oncotarget.12652)
Supplement: Supplementary file 1 [file oncotarget-07-82820-s001.pdf]

Eriocalyxin B, a natural diterpenoid, inhibited VEGF-induced angiogenesis and diminished angiogenesis-dependent breast tumor growth by suppressing VEGFR-2 signaling

SUPPLEMENTARY FIGURES AND TABLES

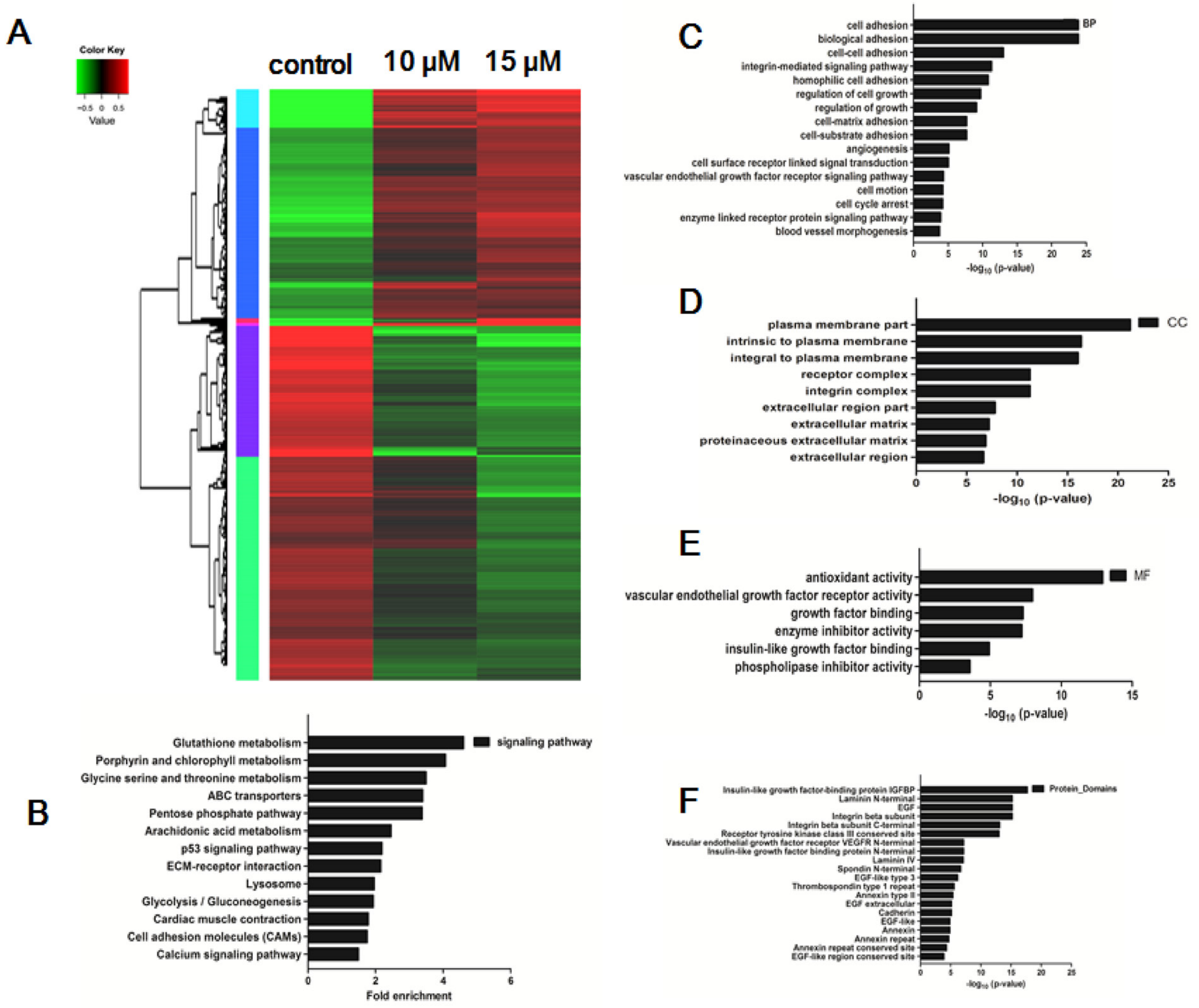

Supplementary Figure S1: Analysis of changed genes, biological pathway and gene ontology involved in EriB exerted anti-angiogenesis. A. Heat map of more than 1500 annotated genes expressions in zebrafish embryos after stimulation with control or EriB (10 and 15 µM) for 72 h (Supplementary Information Table S2). The up-regulated mRNA expression in treated group with respect to control was represented by red colour and down-regulated mRNA expression was presented as green colour. The scale of color intensity was positively correlated to the fold change. **B.** List of signaling pathways involved in the regulation of SIVs formation in zebrafish by EriB. Gene ontology of the down-regulated and up-regulated biological processes **C.** cellular components **D.** molecular function **E.** related to the alteration of SIVs formation in zebrafish by EriB. Prediction of protein-protein interactions **F.** affected by EriB in zebrafish.

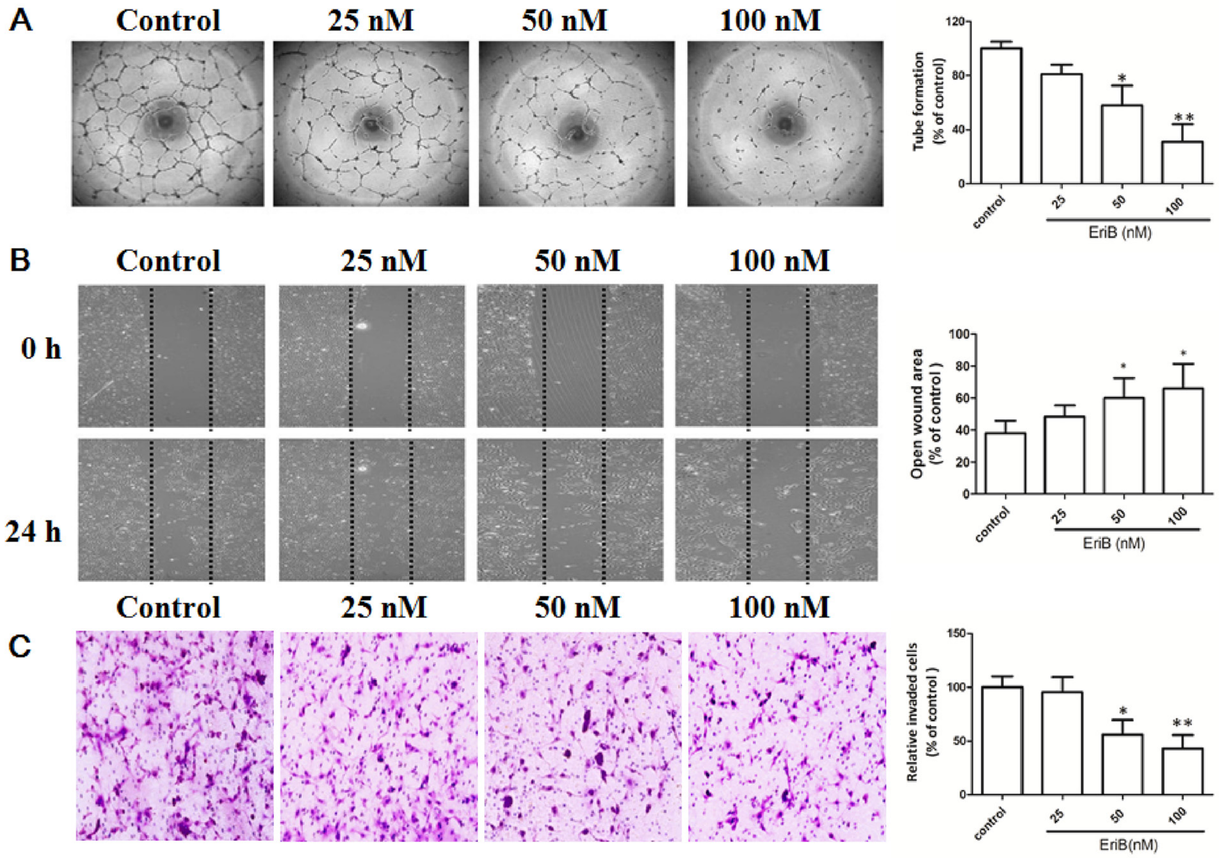

Supplementary Figure S2: EriB inhibited *in vitro* angiogenesis in HUVECs. A. EriB inhibited the tube formation after the incubation for 11 h and tube structures of HUVECs were photographed (magnification, ×40). **B.** EriB suppressed the cell migration after the incubation for 24 h and the wounded area of each well was captured at 0 h and 24 h of incubation (magnification, ×40). **C.** EriB attenuated cell invasion after 11 h incubation, and the migrated cells on the lower side of membranes were stained and counted (magnification, ×100). Each value was presented as means + SD (n=3). * $p < 0.05$, ** $p < 0.01$ compared with control (one-way ANOVA).

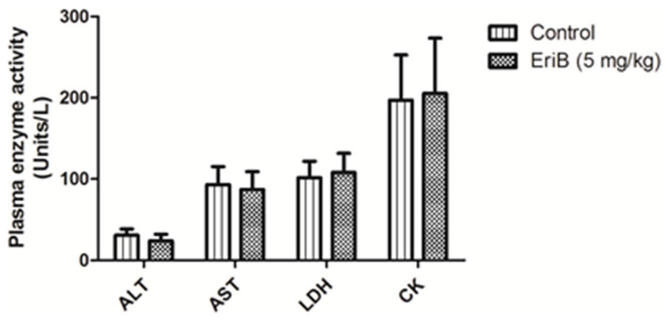

Supplementary Figure S3: EriB exerted no change on the plasma enzymes activities. (A) The plasma enzyme activities of ALT, AST, LDH, and CK in 4T1-bearing mice after 21 days of treatment were detected. Each value was presented as means + SEM (n=6).

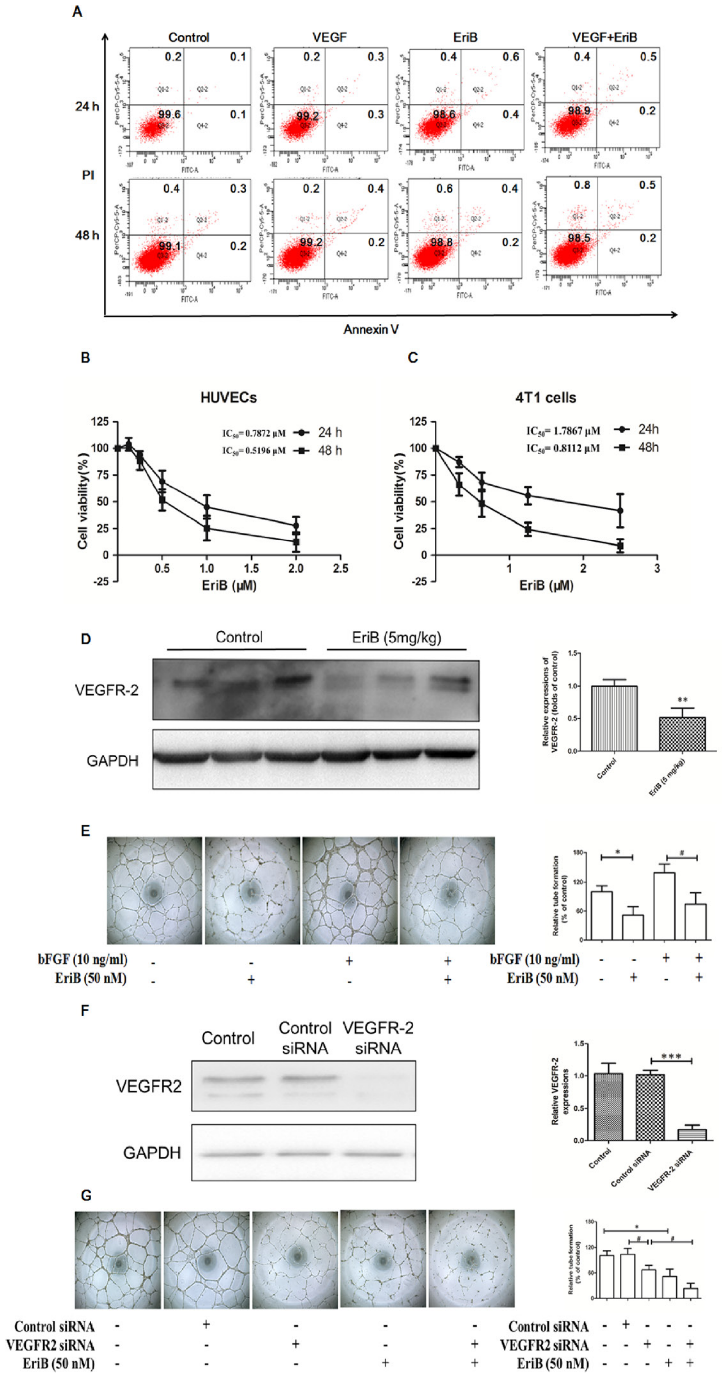

Supplementary Figure S4: Effects of EriB on HUVECs. A. EriB (100 nM) were added to HUVECs in the presence of VEGF (10 ng/mL) for 24 and 48 h. Then cells were collected and subjected to Annexin V-FITC/propidium iodide (PI) double staining and analyzed by flow cytometry. MTT assay was performed to detect the effect of EriB on cell viability when HUVECs **B.** and 4T1 cells **C.** were treated with different concentrations of EriB for 24 and 48 h. Each value was presented as means + SD (n=5). IC_{50} values were calculated with GraphPad Prism. **D.** Tumor proteins were extracted and subjected to western blot for the detection of VEGFR-2 levels. Each value was presented as means + SD (n=3). ** $p < 0.05$ compared with control group. **E.** EriB suppressed the bFGF-induced tube formation after the incubation for 11 h and tube structures of HUVECs were photographed (magnification, ×40). * $p < 0.05$ compared with control, # $p < 0.05$ compared with bFGF group. **F.** HUVECs were treated with negative siRNA (50 nM), VEGFR2 siRNA (50 nM) or only transfection reagent using lipofectamine® RNAiMAX for 48 h, and then whole cells were extracted for western blotting analysis to detect the VEGFR-2 level. *** $p < 0.001$, compared with control. **G.** After having confirmed the VEGFR-2 knockdown efficacy, cells transfected with either VEGFR-2 siRNA, negative siRNA or no siRNA were collected and subjected to tube formation assay. After the incubation for 11 h and tube structures of HUVECs were photographed (magnification, ×40). * $p < 0.05$ compared with control, # $p < 0.05$ compared among groups (one-way ANOVA).

Supplementary Table S1: The identified 72 different angiogenic genes expressions

See Supplementary File 1

Supplementary Table S2: Functional analysis of all the changed genes

See Supplementary File 2
